# A successful approach for angiosarcoma of the scalp using helical tomotherapy and customized surface mold brachytherapy: A case report

**DOI:** 10.1097/MD.0000000000028210

**Published:** 2021-12-10

**Authors:** Gen Suzuki, Koji Masui, Sho Watanabe, Hideya Yamazaki, Tadashi Takenaka, Jun Asai, Ayano Maruyama, Kei Yamada

**Affiliations:** a Department of Radiology, Kyoto Prefectural University Graduate School of Medical Science, Kamigyo-ku, Kyoto, Japan; b Department of Dermatology, Kyoto Prefectural University Graduate School of Medical Science, Kamigyo-ku, Kyoto, Japan.

**Keywords:** angiosarcoma, brachytherapy, case report, helical tomotherapy, IMRT

## Abstract

**Rationale:**

Angiosarcoma of the scalp (ASS) is a rare solid tumor with a high risk of local recurrence. Effective treatment strategies are not currently available for angiosarcoma of the scalp (ASS). The aim of this study was to report the utility of high-dose-rate brachytherapy (HDRBT) as a boost treatment for ASS following total scalp irradiation using helical tomotherapy (HT). This is the first report of successful treatment of ASS using HT and HDRBT.

**Patient concerns:**

An 81-year-old woman presented with hemorrhagic nodular skin tumors of the scalp. The patient first noticed the scalp mass 3 months before consultation, which became significantly enlarged within a short period. The tumor was positioned mostly in the parietal area, although the skin color change was widely spread to the surrounding scalp.

**Diagnosis:**

The patient underwent biopsy of the skin lesion at the right parietal region, which revealed the presence of angiosarcoma on pathological examination. There was neither regional lymphadenopathy nor distant metastases on PET/CT.

**Interventions:**

Considering the patient's old age and poor performance status because of a history of cerebral infarction, we considered that she was eligible for definitive chemoradiotherapy of the scalp. We adopted an individual surface mold HDRBT boost of 18 Gy in three fractions following total scalp irradiation with 50 Gy in 25 fractions delivered using HT. Docetaxel (40 mg/m^2^) was administered every 4 weeks, concurrently with radiotherapy.

**Outcomes:**

Treatment tolerance was good, and severe toxicity has not been observed to date. At 18 months after radiotherapy, the patient does not have any evidence of recurrence.

**Conclusion:**

Customized surface mold HDRBT following total scalp irradiation using HT resulted in excellent disease control and minimal toxicity; thus, it may be a promising therapeutic option for ASS.

## 1 Introduction

Angiosarcoma of the scalp (ASS) is a rare solid tumor with a high risk of local recurrence. Radiation therapy represents a valuable curative therapeutic option, as radical surgery is rarely feasible because of local spread and the multicentric nature of this cancer type.^[1]^ Recent advances in external irradiation equipment have provided several new cancer radiation therapy modalities, such as intensity-modulated radiation therapy (IMRT), which may play an important role in the total scalp irradiation.^[2–5]^ Several recent studies have compared IMRT and/or helical tomotherapy (HT) with the older electron or lateral photon electron (LPE) technique, and have demonstrated improved homogeneity, better conformality and reduced doses to the brain that favored HT.^[3–6]^

High-dose-rate brachytherapy (HDRBT) based on a mold is another option to treat ASS, although few reports of this technique are available.^[7–10]^ Brachytherapy theoretically enables the delivery of higher doses to the scalp surface and lower doses to deep-seated areas compared with external beam radiotherapy. Thus, brachytherapy may be an optimal treatment modality for ASS. However, HDRBT has the drawback of the high dose delivered to critical organs at risks (OARs), such as the brain and eyes.^[5,11]^ These published studies indicate that the combination of HT and HDRBT may be a promising treatment modality for ASS.

In this report, we present the first case of ASS that was successfully treated with combined HT and individual surface mold HDRBT.

## 2 Case report

**Figure 1:**
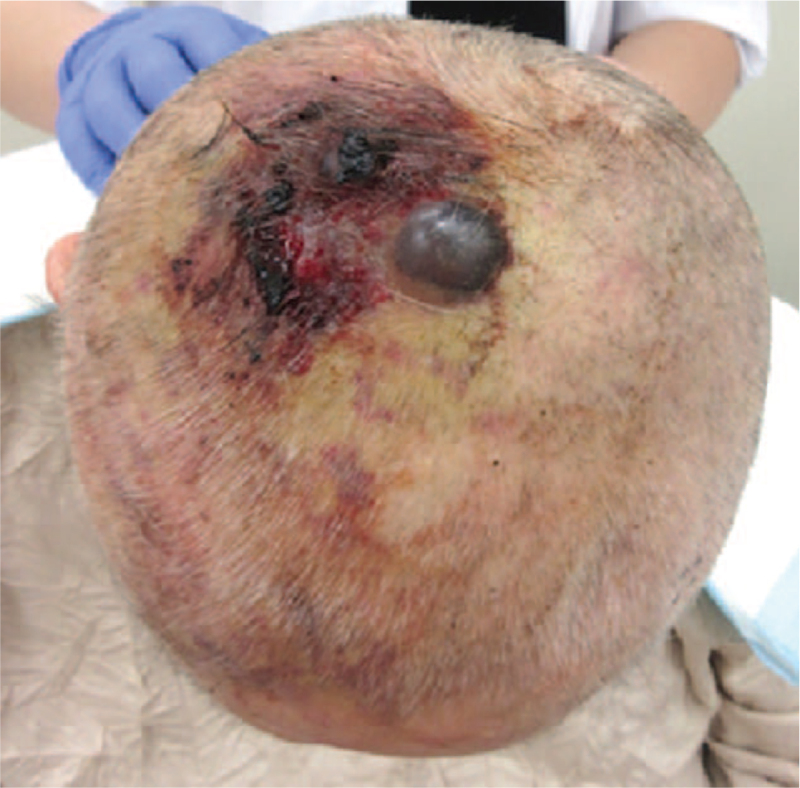
Macroscopic appearance of the patient's angiosarcoma of the scalp before the treatment.

An 81-year-old woman presented with hemorrhagic nodular skin tumors of the scalp. She had a past medical history of cerebral infarction at the age of 76-year-old. Her social and family history was unremarkable. Her environmental history revealed no abnormalities. The patient first noticed the scalp mass 3 months before consultation, which became significantly enlarged within a short period. The tumor was positioned mostly in the parietal area, although the skin color change was widely spread to the surrounding scalp (Fig. 1). The patient underwent biopsy of the skin lesion at the right parietal region, which revealed the presence of angiosarcoma on pathological examination. Positron emission tomography/computed tomography (PET/CT) scanning was performed to determine the exact extent of the lesion; the main tumor had a maximum thickness of 14 mm and a width of 53 mm at the largest diameter. There was neither regional lymphadenopathy nor distant metastases on PET/CT. Considering the patient's old age and low performance status equal to Karnofsky scale index of 60% because of a history of cerebral infarction, we considered that she was eligible for definitive chemoradiotherapy of the scalp, followed by adjuvant chemotherapy. Informed consent was obtained from the patient and her family.

### 2.1 Helical tomotherapy

**Figure 2:**
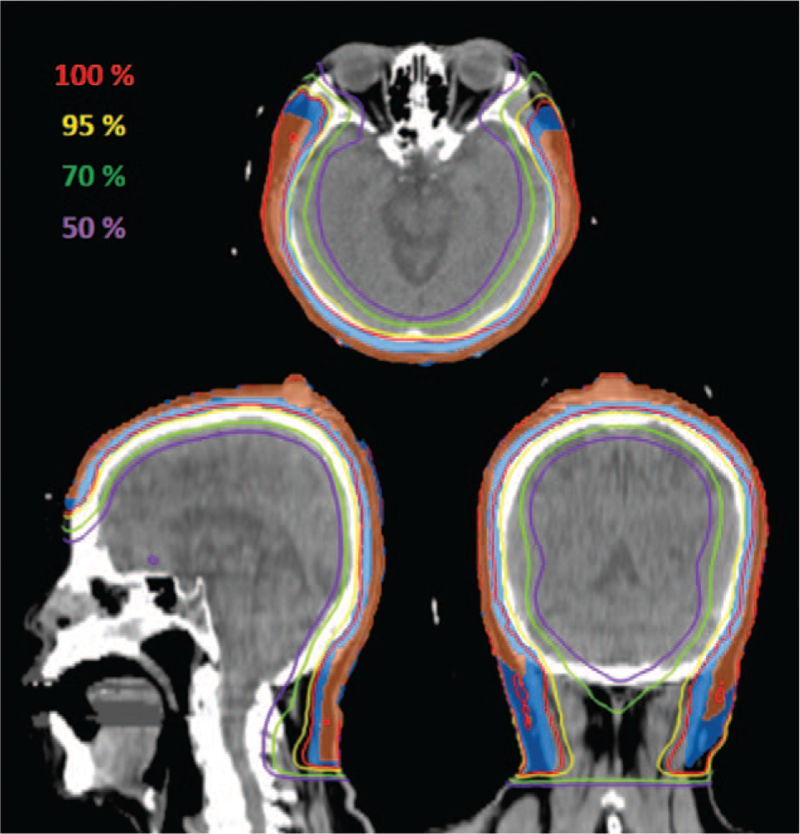
Dose distributions of helical tomotherapy. Axial, coronal, and sagittal views of computed tomography with superimposed dose distribution. The clinical target volume and planning target volume are rendered in orange and blue, respectively.

We decided to deliver a total dose of 50 Gy in 25 fractions to the total scalp using an HT delivery system (Radixact: Accuray Incorporated, Sunnyvale, CA) with 6-MV X-rays. To reduce the risk of scalp recurrence, we used skin boluses placed on the whole scalp, to minimize the skin-sparing effect of high-energy photons. The planning CT scan was obtained using a 2 mm slice thickness with the patient wearing a head-and-shoulder thermoplastic mask. The treatment plan was created using the Precision ver 2.0.1.1 software (Accuray Incorporated, Sunnyvale, CA). The clinical target volume (CTV) was defined as the total scalp. The planning target volume (PTV) was determined by adding an isotropic 5 mm margin to the CTV. The OARs (brain, eyes, optic nerves, chiasm, and lenses) were subsequently contoured. HT plans were normalized to D95%PTV = 100% of the prescription dose (50 Gy in 25 fractions). The minimum, maximum, and mean doses to the PTV were 39.21, 53.5, and 51.03 Gy, respectively. The maximum doses (D0.1cc) for the OARs were 50.19 Gy to the brain, 17.18 Gy to the eyes, 20.79 Gy to the optical nerves, 10.81 Gy to the chiasm, and 8.54 Gy to the lenses. The dose distributions obtained are shown in Figure 2, which illustrates that it was possible to treat the entire scalp with significant sparing of the adjacent OARs.

A megavolt CT scan was performed prior to each fraction, to ensure optimal target coverage and setup accuracy. Docetaxel (40 mg/m^2^) was administered every 4 weeks, concurrently with radiotherapy. Through clinical examination during the course of treatment, we confirmed that the size of scalp lesions was significantly reduced. Considering the patient's good compliance, we planned for HDRBT using a customized surface mold as a boost treatment.

### 2.2 Brachytherapy

**Figure 3:**
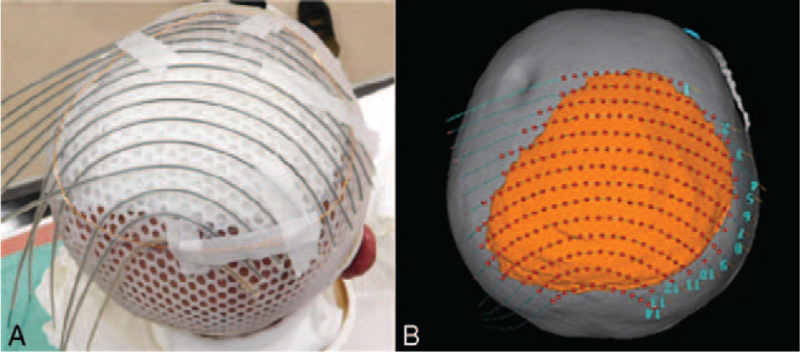
(A) Customized mold applicator. (B) Association of radiation source arrangement.

An area beyond the gross tumor with 2 cm margins was marked over the skin as the CTV. A standard thermoplastic mask designed to conform to the unique topology of the patient's scalp was prepared, to serve as the base for applicator attachment. The target area was redrawn over the mask. To ensure proper dose delivery on the borders of the target, 14 plastic catheters was selected to cover the whole target area including the margin, and they were taped to the mold with a spacing of ∼1.0 cm (Fig. 3). We used 14 catheters to treat the lesion. After completion of this procedure, a CT study of 2 mm thickness was performed to plan the radiotherapy.

**Figure 4:**
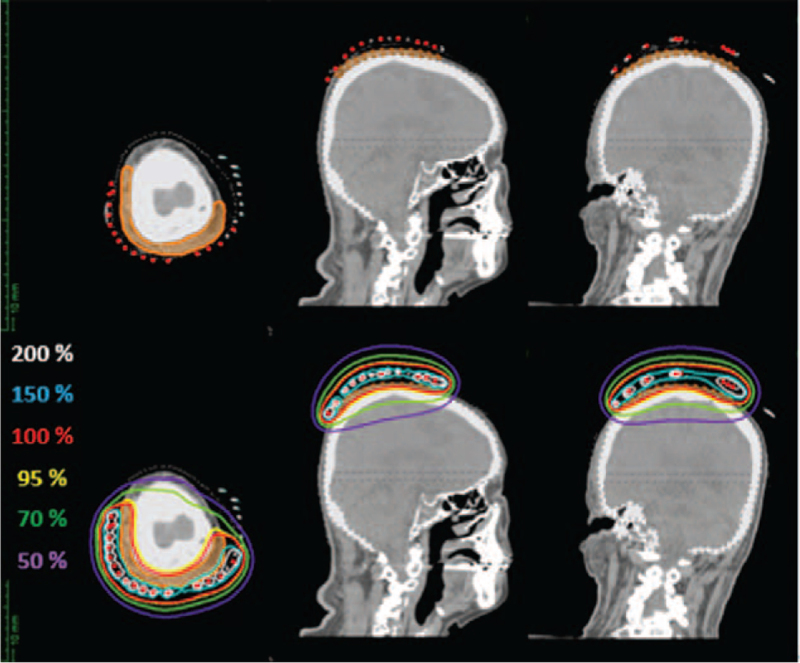
Reconstruction of catheters and dose distribution in boost brachytherapy. The clinical target volume is shown in orange.

The OncentraBrachy treatment planning system (Elekta AB, Stockholm, Sweden) was used to simulate three-dimensional dose distribution. After image reconstruction, the CTV corresponding to the target area and the OAR (brain) was contoured on the axial slices. The skin and the soft tissue of the scalp formed the CTV. The CTV was delineated according to the wire markers visible on the CT scan, which was located along the target area. Dose points were created around the catheters, as shown in the figure (Fig. 3). Following this initial step, volumetric optimization was performed to make the 100% prescription isodose conform to 95% of the CTV volume. We took care to reduce the volume of the target covered by the 150% isodose curve as much as possible. The total prescribed dose was 18 Gy delivered every other day in three fractions of 6 Gy. HDRBT was carried out using a ^192^Ir remote after-loading system (MicroSelectron v3 HDR, Nucletron, ELEKTA AB, Stockholm, Sweden). Dose distribution to the target and brain was also analyzed on the CT scans (Fig. 4). The maximum dose (D0.1cc) and D2cc for the brain were 14.68 and 13.60 Gy, respectively

### 2.3 Follow-up and toxicity assessment

**Figure 5:**
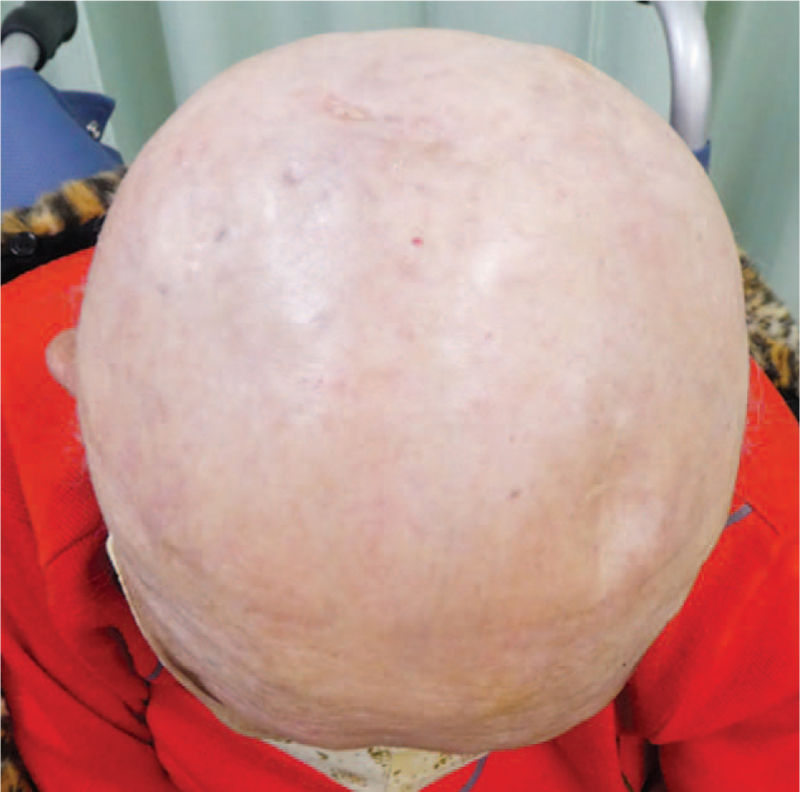
Macroscopic appearance of the patient's angiosarcoma of the scalp 18 months after radiotherapy.

No severe acute complications occurred during the treatment, and the radiotherapy was well tolerated. The patient developed a maximum of Grade II dermatitis as an acute adverse event, which was successfully managed symptomatically. At the 2-month follow-up after HDRBT, the dermatitis had improved markedly and there was good clinical response with no residual disease. In addition, good cosmesis was achieved. At the 18-month follow-up, visual inspection and radiological studies showed no recurrence, and good overall cosmesis was observed (Fig. 5). The patient has received adjuvant chemotherapy with docetaxel every 4 weeks and has been kept on follow-up.

## 3 Discussion

Here, we reported the use of customized surface mold HDRBT as boost radiotherapy in a case of ASS with good local control. To the best of our knowledge, this is the first report of successful treatment of ASS using HT and HDRBT.

The treatments of choice for ASS, including surgery, radiation therapy, and chemotherapy, have evolved in the last few decades.^[2,6,12–16]^ In particular, irradiation techniques for the treatment of ASS have changed dramatically, which would have an impact on the treatment results. Scalp irradiation represents a dosimetric challenge because of the irregular shape of the target and the difficulty in obtaining a homogeneous dose distribution while sparing the nearby critical structures. For many years, LPE techniques were the first choice for treating scalp sites, because of their superficial distribution and rapid falloff. Electron therapy should be delivered perpendicular to the skin's surface; however, the irregular shape of the skull results in a nonuniform dose distribution, with areas of hot/cold spots. In an attempt to address this problem, the advanced technique of IMRT is currently being utilized at some institutions for total scalp irradiation.^[3–5,11]^ In particular, several clinical studies of rotational IMRT, which is typified by HT and volume-modulated radiotherapy (VMAT), have reported an excellent, and homogeneous dose distribution over the whole scalp, while reducing the irradiation dose and volume given to OARs, such as the brain.^[2–4]^ Song et al compared LPE, HT, and VMAT plans for total scalp irradiation and concluded that the HT plan showed the best target coverage and conformity, with low doses to the brain, especially the hippocampus.^[4]^ Another advantage of HT is its increased safety regarding treatment delivery, because all target volumes are aligned and verified daily, prior to each fraction, based on megavolt CT scans.

Guadagnolo et al reported that approximately two-thirds of patients experienced local recurrence in an analysis of 70 patients with angiosarcoma. The authors concluded that local recurrence was associated with a high mortality rate and that local disease control was crucial.^[14]^ The efficacy of radiation dose escalation for the local treatment of ASS has been reported.^[13,17]^ Brachytherapy is a promising radiation delivery technique, considering its excellent conformality for dose escalation. Its use allows for biologically efficient dose escalation within the tolerance of the surrounding OARs.^[5]^ However, the data on HDRBT in the patients with ASS are scarce, as they are limited to very small clinical trials and some case reports.^[7–10]^

In the dosimetric analysis of an LPE plan with IMRT and HDRBT reported by Wojcicka et al, HDRBT was shown to be the most conformal plan, but the total dose delivered was limited because of the brain and optic structures. The authors suggested that HDRBT is a clinically suitable treatment for less extensive lesions and lower prescription doses^[5]^; that is, HDRBT may theoretically be the optimal boost treatment for ASS because it allows conformal planning. Despite its total dose limitations, the HDRBT technique has several advantages:

1. the treatment delivery is simple,
2. the setup is highly reproducible,
3. the treatment time is short, and, thus,
4. the treatment is well tolerated.

Therefore, HDRBT should be considered as a useful technique for ASS management, and customized surface mold HDRBT may be beneficial in the individualized approach.

It is well known that ASS is locally aggressive, and the vascular nature of the scalp places large portions of the scalp at risk of developing disease. Our center adopted an HDRBT boost of 18 Gy in three fractions to follow a total scalp irradiation of 50 Gy in 25 fractions delivered using HT. However, sufficient data to determine the optimal and most effective radiation regimen are lacking. In addition, the technical conditions at specific institutions might limit the scope of choice of possible techniques.

The apparent limitation of our case report is that we were able to evaluate only one patient, which limits the ability to extrapolate the current results to other cases. Nonetheless, we believe that our report adds to the current literature on, and clinical experience with HDRBT as an effective modality that may lead to beneficial outcomes.

## 4 Conclusion

This was the first report of successful treatment of ASS using IMRT and HDRBT. Our findings showed that customized surface mold HDRBT following total scalp irradiation using HT resulted in excellent disease control and minimal toxicities, and may be a promising therapeutic option for ASS.

## Author contributions

**Conceptualization:** Gen Suzuki, Koji Masui.

**Investigation:** Sho Watanabe, Tadashi Takenaka.

**Methodology:** Koji Masui, Tadashi Takenaka.

**Resources:** Jun Asai, Ayano Maruyama, Koji Masui.

**Supervision:** Hideya Yamazaki.

**Writing – original draft:** Gen Suzuki, Koji Masui.

**Writing – review & editing:** Gen Suzuki, Kei Yamada.
